# *Flavivirus* infections induce a Golgi stress response in vertebrate and mosquito cells

**DOI:** 10.1038/s41598-021-02929-1

**Published:** 2021-12-06

**Authors:** Mercedes Viettri, José L. Zambrano, Romel Rosales, Gerson I. Caraballo, Ana Lorena Gutiérrez-Escolano, Juan E. Ludert

**Affiliations:** 1 Department of Infectomics and Molecular Pathogenesis, Center for Research and Advanced Studies (CINVESTAV-IPN), Mexico City, Mexico; 2 Center of Microbiology and Cell Biology, Venezuelan Institute for Scientific Research (IVIC), Caracas, Venezuela; 3 Present Address: Department of Microbiology, Mount Sinai School of Medicine, NY, USA

**Keywords:** Pathogens, Virology, Microbiology

## Abstract

The stress of the Golgi apparatus is an autoregulatory mechanism that is induced to compensate for greater demand in the Golgi functions. No examples of Golgi stress responses due to physiological stimuli are known. Furthermore, the impact on this organelle of viral infections that occupy the vesicular transport during replication is unknown. In this work, we evaluated if a Golgi stress response is triggered during dengue and Zika viruses replication, two flaviviruses whose replicative cycle is heavily involved with the Golgi complex, in vertebrate and mosquito cells. Using GM-130 as a Golgi marker, and treatment with monensin as a positive control for the induction of the Golgi stress response, a significant expansion of the Golgi cisternae was observed in BHK-21, Vero E6 and mosquito cells infected with either virus. Activation of the TFE3 pathway was observed in the infected cells as indicated by the translocation from the cytoplasm to the nucleus of TFE3 and increased expression of pathway targeted genes. Of note, no sign of activation of the stress response was observed in CRFK cells infected with Feline Calicivirus (FCV), a virus released by cell lysis, not requiring vesicular transport. Finally, dilatation of the Golgi complex and translocation of TFE3 was observed in vertebrate cells expressing dengue and Zika viruses NS1, but not NS3. These results indicated that infections by dengue and Zika viruses induce a Golgi stress response in vertebrate and mosquito cells due to the increased demand on the Golgi complex imposed by virion and NS1 processing and secretion.

## Introduction

The endoplasmic reticulum (ER) and the Golgi apparatus are two tightly connected organelles that are key in the synthesis, processing, and secretion of eukaryotic cells proteins. These organelles are bound by membranes that allow for the compartmentalization of specialized cellular functions. Upon an excessive, overwhelming demand of the functions of the ER or the Golgi apparatus, these organelles respond by a mechanism of homeostatic autoregulation termed stress responses, aimed to augment their functional capacity^1–3^.

Viral infections are inductors of stress in the infected cell. In particular, flavivirus replication processes leading to the production of progeny virions, interfere with a number of aspects of the cellular metabolism, triggering a homeostatic disequilibrium^4,5^. The flavivirus genome is a single stranded positive sense RNA molecule of approximately 10 kb in length, which encodes one large polyprotein that, after proteolytic processing by host and viral proteases, gives rise to the three structural proteins (capsid, membrane precursor, and envelope) and seven no structural proteins (NS1, NS2A, NS2B, NS3, NS4A, NS4B and NS5) that play essential roles in genome replication, polyprotein processing and virion assembly. Translation and replication of the flavivirus genome, as well as virion formation are all processes that occur in the ER and involve extensive ER membrane modifications^6–9^. Consequently, ER stress and the activation of the Unfolded-Protein-Response (UPR) have been well documented in vertebrate and mosquito cells infected with dengue (DENV) or Zika (ZIKV) viruses^5,10–13^.

After leaving the ER complex, immature virions enter the Golgi apparatus and following a classical secretory pathway, are transported to the plasma membrane to be released out of the cell by exocytosis. During their transit thru the Golgi complex, immature virions undergo glycan modifications in the envelope (E) protein and proteolytic cleavage of the membrane precursor protein (M), by action of the Golgi resident protease furin, to become mature infectious virions^14–16^. In addition, DENV and ZIKV infected vertebrate and mosquito cells, also secrete the NS1 protein as a hexamer. In vertebrate cells, the NS1 protein is released following a classical secretory pathway using the Golgi apparatus, where the N-glycans attached to NS1 suffer further processing^5,6,8,17^. At variance, in mosquito cells, the NS1 protein is secreted using a non-classical secretory pathway in association with the chaperone caveolin complex^17,18^. All those secretory processes may impact and overwhelm the capacity of the Golgi.

The Golgi apparatus is an organelle with various functional zones, including the cis-Golgi network, cis-Golgi, medial-Golgi, trans-Golgi, trans-Golgi network, separated by membranes and distinct cisterns, where diverse cellular functions such as glycosylation, sulfation, fucosylation and phosphorylation of secretory and membrane proteins is carried out^2,3^. In the cells that need a greater demand for the functions of Golgi, as could be the case of flaviviruses infected cells, the capacity of this organelle collapses. To compensate this functional demand a mechanism of autoregulation homeostatic is induced, which is termed Golgi stress response which involve several activation or response pathways^1–3,19^.

In this work, we explore if the high involvement of the Golgi complex during flavivirus replication results in the induction of the Golgi stress response. Our results indicate that DENV and ZIKV infection activates the Golgi stress response in both vertebrate and mosquito cell lines. Moreover, the sole expression of the NS1 protein can induce the stress response in vertebrate cells. Interestingly, the replication of feline calicivirus, a virus not involved with the Golgi apparatus, do not result in a stress response. The understanding of the activation and regulation of the Golgi stress response is still incomplete^3^; our results suggest that flavivirus infections can become an additional tool to study this phenomena.

## Results

### Flavivirus infection results in a Golgi stress response in both vertebrate and mosquito cells

**Figure 1:**
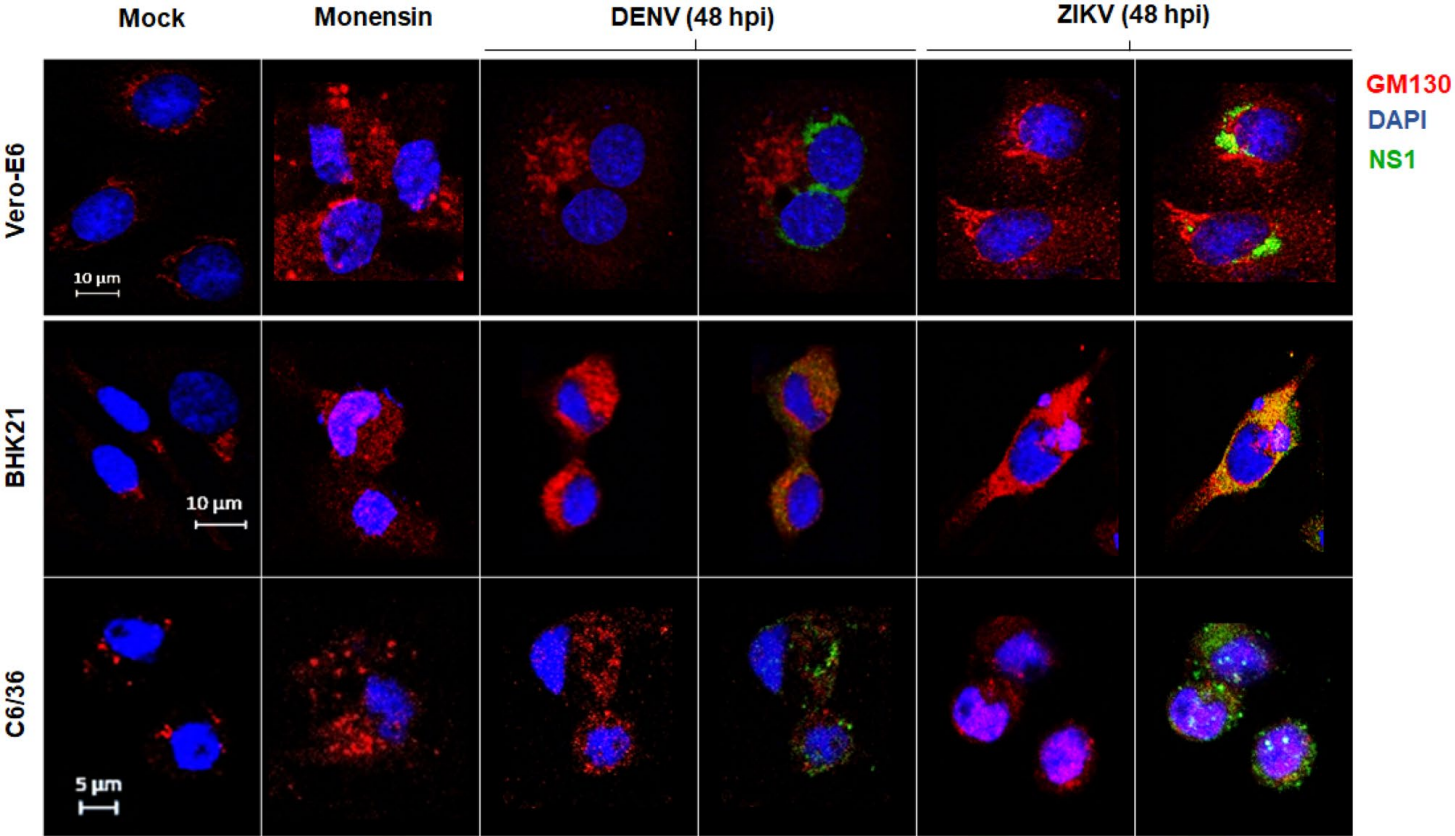
Golgi stress response due to flavivirus infection in vertebrate and mosquito cells. Cells were either treated with monensin and fixed at 24 h or infected with DENV and ZIKV at MOI = 3 and fixed at 48 hpi. Cells were probed against flavivirus NS1 (green), and GM130 (cis-Golgi, red). Nuclei were counter stained with DAPI (blue). Merged fluorescent images of DAPI, red and green channels are shown. The images were analyzed using a Zeiss LSM 700 confocal microscope with laser sections: 0.45 μm. Images from one out of least 3 independent experiments are shown.

The Golgi stress response is a consequence of an increased demand on the organelle functions. Given that flavivirus replication and NS1 and virion secretion are processes heavily dependent on the Golgi apparatus, we tested if a Golgi stress response is induced in cells infected with DENV or ZIKV. As a positive control, the Na^+^/H^+^ ionophore monensin was used at non-toxic concentrations. Monensin is an agent that blocks glycosylation and vesicular transport of proteins and induce Golgi-stress response in treated cells^3,20^. Golgi expansion or fragmentation was visualized using GM-130 as a marker. In mock infected cells, GM-130 mark was observed a dotted staining near the nuclei of the cell, that correspond to the reporter Golgi subcellular localization (Fig. 1). Monensin treatment results in a fuzzy pattern of GM-130 staining throughout all the cytoplasm, indicating fragmentation and dispersion of the Golgi apparatus in treated cells (Fig. 1). A pattern like the one obtained with monensin treatment was observed in the Vero, BHK-21 and C6/36 cells infected with DENV and ZIKV at 48 h post-infection (Fig. 1). These results suggest that DENV and ZIKV replication results in a Golgi stress response in vertebrate as well as mosquito cells.

### DENV and ZIKV infection in vertebrate cells results in the activation of the transcription factor E3 (TFE3) pathway

**Figure 2:**
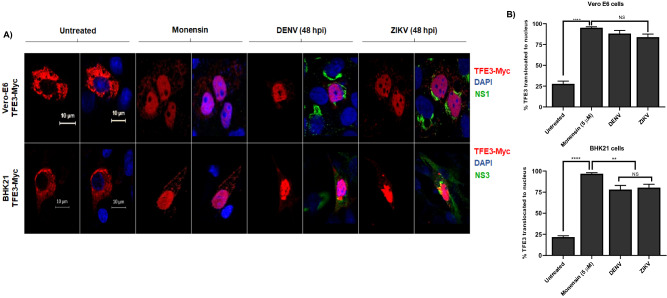
Activation of TFE3 pathway due flavivirus infection in vertebrate cells. (**A**) Cells lines Vero-E6 and BHK-21 were transfected with 1 µg of plasmid DNA of transcriptional factor TFE3 (TEF3-Myc). Twenty-four h after transfection, cells were treated with monensin and fixed 24 h after treatment or infected with DENV and ZIKV and fixed 48 hpi. Cells were probed against flavivirus NS1 (green), and Myc (TFE3, red). Nuclei were counter stained with DAPI (blue). Merged fluorescent images of DAPI, red and green channels are shown. The images were analyzed using a LSM 700 confocal microscope with laser sections: 0.45 μm. Images from one out of least 3 independent experiments are shown. (**B**) Percentage of TFE3 translocated from the cytoplasm to nucleus in both cells lines were measured through images analysis with Zen Blue edition 2.6 software and the results were plotted using GraphPAD Prism version 8.01. *p ≤ 0.05.

One of the pathways activated during the Golgi stress response is the TFE3 pathway. TFE3 located in the cytoplasm, is dephosphorylated, and translocated to the cell nuclei upon activation of the Golgi stress response. To corroborate the activation of the stress response, vertebrate cells transfected with a recombinant form of TFE3 were infected with DENV and ZIKV. As shown in Fig. 2A, in untreated cells the mark corresponding to TFE3 is located mainly in the cytoplasm. However, monensin treatment or viral infections resulted in a significant translocation of TFE3 from the cytoplasm to the cell nuclei. In Vero-E6 cells, a statistically significant difference was observed in the percentage of TFE3 translocation between the mock condition (27.8%) and cells treated with monensin (95.2%) or cells infected with DENV (88.2%) or ZIKV (83.9%). A similar effect was observed in BHK 21 cells, with a percentage of TFE3 translocation of 21.7% in untreated cells, 96.8% in cells treated with monensin, 78% in DENV infected cells and 80.4% in ZIKV infected cells (Fig. 2B). These results indicate that the TFE3 pathway is activated in vertebrate cells infected with DENV and ZIKV. Since no expression of TFE3 was observed in transfected mosquito cells, C6/36 were not considered in this experiment.

**Figure 3:**
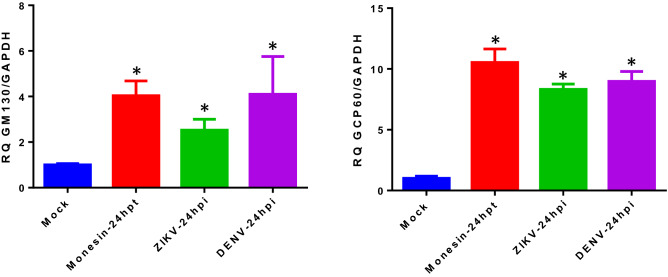
Increase expression of genes targeted by the TFR3 pathway in infected cells. BHK-21 cells were infected with DENV and ZIKV using a MOI = 3 and harvested at 24 hpi. Mock infected and monensin treated cells were included as negative and positive controls, respectively. Changes in GOLGA 2 (GM130) and GCP60 gene expression levels were quantified by qRT-PCR using the delta-delta Ct method and GAPDH as housekeeping gene. Results are the mean ± SD of two independent experiments. Differences in mRNA expression levels were analyzed for statistical significance using the ANOVA test. *p ≤ 0.05.

To corroborate the activation of the TFE3 pathway and Golgi stress response in DENV and ZIKV infected cells, the expression of two genes targeted by this pathway was evaluated by qRT-PCR in infected BHK-21 cells, harvested at 24 hpi. Figure 3 shows that upon infection with DENV or ZIKV, the mRNA levels of the GOLGA2 and GCP60 genes, encoding respectively for the structural GM130 and GCP60 proteins, were significantly increased in relation to mock infected cells, and to levels comparable with those observed in monensin treated cells. These results confirm the activation of the TFE3 pathway upon infection.

### FCV infection do not activate the Golgi stress response

**Figure 4:**
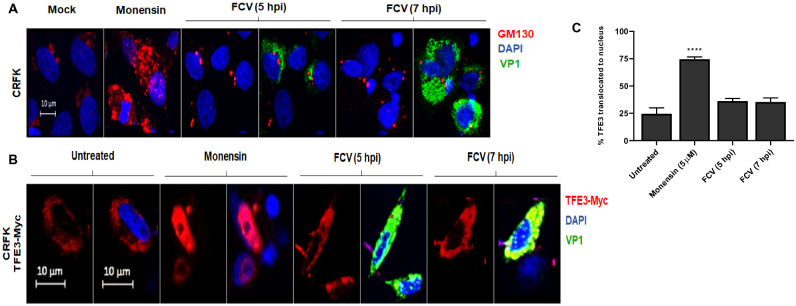
CRFK cells infected with FCV showed no evidence of Golgi stress or activation of the TFE3 pathway. (**A**) CRFK cells were infected with FCV and fixed at 5 and 7 hpi. The cells were probed against capsid protein VP1 (green), and GM130 (cis-Golgi, red). Nuclei were counter stained with DAPI (blue). (**B**) CRFK cells were transfected with 1 µg of plasmid DNA of transcriptional factor TFE3 (pTEF3-Myc), then infected with FCV at MOI = 5 and fixed at 5 and 7 hpi. Cells were probed against capsid protein VP1 (green), Myc (TFE3, red). Nuclei were counter stained with DAPI (blue). Cells were analyzed using a Zeiss LSM 700 confocal microscope with laser sections: 0.45 μm. (**C**) Percentage of TFE3 translocated from the cytoplasm to nucleus were measured through images analysis with Zen Blue edition 2.6 software and the results were plotted using GraphPAD Prism version 8.01. *p ≤ 0.05.

FCV is a highly lytic virus, whose replication cycle have little or no involvement with the Golgi apparatus^21^. Thus, to address if the observed activation of the Golgi stress response was specific for DENV and ZIKV infections, CRFK cells infected with FCV were analyzed for Golgi expansion or translocation of the TFE3 factor. CRFK cells were chosen because they are highly permissive to FCV and will magnify any putative use of the Golgi complex. GM-130 staining of mock infected CRFK cells was observed as a dotted mark near the nuclei. In addition, TFE3 was observed in the cytoplasm of untreated cells. No changes in the architecture of the Golgi complex or translocation of the TFE3 factor to the cell nuclei were observed in infected CRFK fixed at 5 and 7 hpi (Fig. 4). Yet, CRFK cells can mount a Golgi stress response as indicated by the results obtained in monensin treated cells, where changes in the GM-130 mark and translocation of TFE3 were observed. These results suggest that demanding of Golgi functions during the virus replicative process of DENV and ZIKV, but not FCV, are necessary for activation of the Golgi stress response.

### Expression of recombinant DENV NS1, but not NS3, results in activation of the Golgi stress response

**Figure 5:**
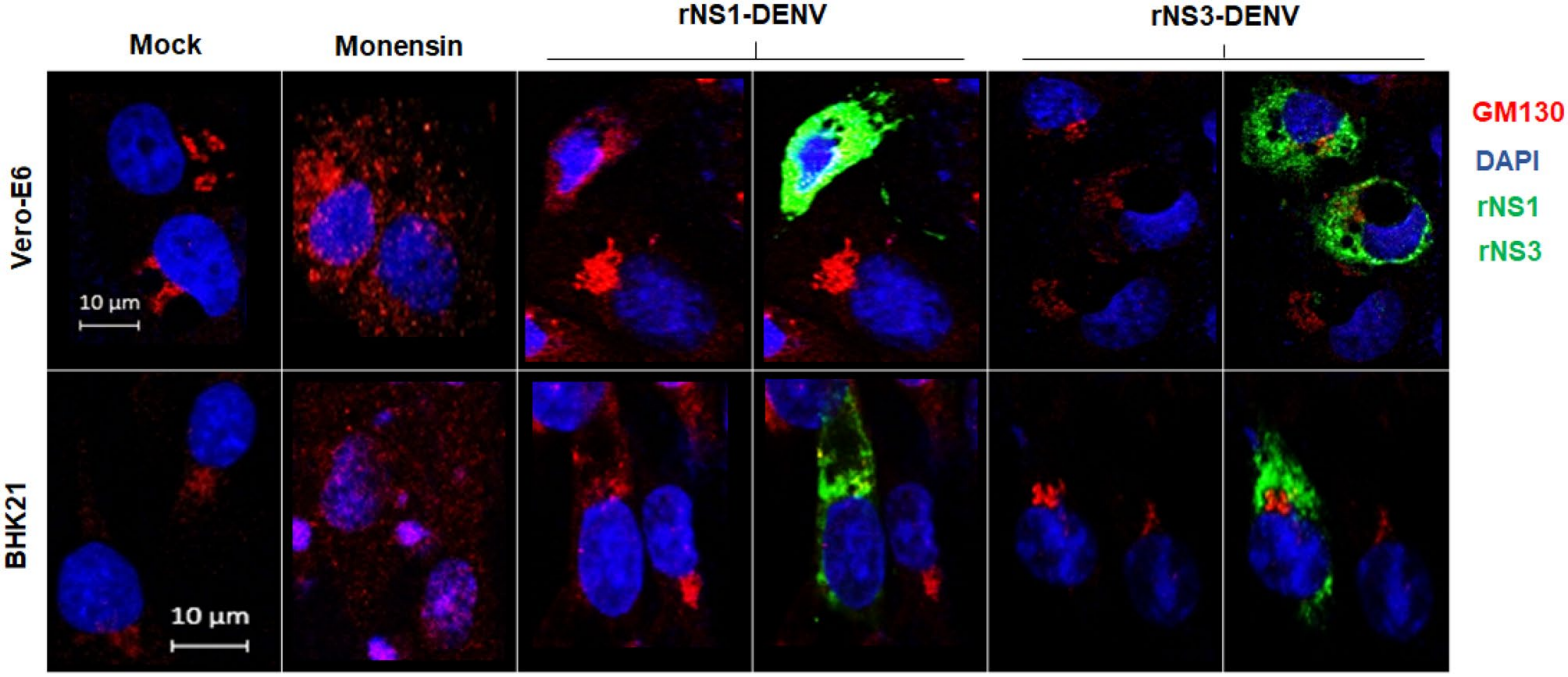
Golgi stress response in vertebrate cells due to expression of recombinant NS1 of dengue virus. Cells were transfected with 1 µg of plasmid DNA of DENV-NS1 and DENV-NS3 or treated with monensin as a positive control and fixed after 24 h. Cells were probed against flavivirus NS1 and NS3 (green), GM130 (cis-Golgi, red). Nuclei were counter stained with DAPI (blue). Merged fluorescent images of DAPI, red and green channels are shown. The images were analyzed using a Zeiss LSM 700 confocal microscope with laser sections: 0.45 μm. Images from one out of least 3 independent experiments are shown.

The secretion of hexameric NS1 in vertebrate cells takes place following the classical secretory route and the Golgi apparatus. To test if the secretion of NS1 alone could trigger the Golgi response, Vero and BHK-21 cells were transfected with a plasmid expressing a recombinant DENV NS1 protein, able to be secreted. Expression of the recombinant NS1 of DENV for 24 h resulted in activation of the Golgi stress response in Vero-E6 and BHK-21 cells, as seen by the diffuse cytoplasmic GM-130 staining in both transfected cells. On the other hand, when the ER resident NS3 protease was expressed, no activation of the response was observed (Fig. 5). These results suggest that the expression and transit of NS1 alone is sufficient to activate the Golgi stress response in vertebrate cells.

## Discussion

The mosquito borne flaviviruses DENV and ZIKV are etiologic agents of important public health diseases affecting tropical and subtropical countries around the globe^22^. The replicative cycle of these and other flavivirus takes place in close contact with membrane bound organelles which are directly or indirectly affected by the viral replicative process. Extensive ER membrane modifications and induction of an UPR, changes in the shape of mitochondria, and even nuclear pore degradation have all been reported in DENV infected cells^13,23,24^. Yet, and despite that the processing and secretion of hexameric NS1 and mature virions in vertebrate cells, as well as the processing and secretion of mature virions in mosquito cells, are all processes that take place in the Golgi apparatus^17^, little is known regarding the response of the Golgi system to flavivirus infections. In this work, evidence obtained in three different cell lines, from vertebrate and mosquito origin, is presented indicating that DENV and ZIKV infections triggers the Golgi stress response and the activation of the TFE3 pathway in infected vertebrate cells. If other mosquito borne flaviviruses not included in this study, such as JEV or WNV, or even the HCV induce a Golgi stress response in infected cells remains to be determined, but given the heavy involvement of all flaviviruses with the Golgi apparatus^7^, this is expected to be the case. Interestingly, the activation of the Golgi stress response seems not to be a generalized phenomenon of viral infections as suggested by the observation that FCV does not trigger these responses in CRFK infected cells, although these cells possess all the molecular mechanisms necessary to go into Golgi stress.

In their transit to the extracellular space, immature DENV and ZIKV virions enter the Golgi apparatus where the E protein suffers trimming of the N-glycan residues^25^ and later the prM protein is proteolytically processed by the Golgi resident acidic protease furin to finally form mature virions^15^. In addition, in vertebrate cells, the secretion of NS1 protein from infected cells utilizes the entire Golgi system, where the two N-glycan residues attached to NS1 undergo further processing^25^. Moreover, the NS1 located on the plasma membrane is GPI-anchored protein, another function that take place in the Golgi cisternae^26^. More recently, multiple components of the Golgi apparatus were found in the interactome of NS1 in vertebrate cells^27^. In this work, we demonstrate that activation of the Golgi stress response occurs also in cells expressing NS1 by itself. So, along with vesicle transport, flavivirus proteins such as E, pr-M and NS1 suffer several post-transcriptional modifications in their transit over the Golgi cisternae. The TFE3 pathway activation ends in the expression of genes involved in augmenting the functions of the Golgi apparatus in general, including Golgi structure, N-glycosylation functions and vesicular transport, rather than a specific function^3^. Thus, the activation of the TFE3 pathway in DENV and ZIKV infected cells is in line with the import and multiple roles played by the Golgi complex during flavivirus replication. Other pathways also activated during the Golgi stress response, such as the CREB3 and the HSP47 pathways conduct to the activation of pro-apoptotic and anti-apoptotic genes^3^. The infection of mouse endothelial cells by HSV-1 causes GM-130 degradation and Golgi apparatus fragmentation that finally leads to cell apoptosis^28^. Both, activation and suppression of apoptosis have been reported in DENV infected cells^29,30^. If the CREB3 or the HSP47 pathways are activated during DENV and ZIKV replication is unknown.

The UPR triggered during flavivirus replication have been linked with mechanisms favoring viral replication such as modulation of innate immunity and induction of autophagy^10,11,29^. The significance of the activation of the Golgi stress response during flavivirus replicative cycle is unclear, but it is reasonable to speculate that augmenting the capacity of the Golgi apparatus will favor the secretion of NS1 and mature virions. Consequently, a better understanding of the interplay between DENV and ZIKV proteins and the Golgi apparatus may help to identify antiviral targets common to vertebrate and mosquito cells.

Finally, we like to remark that several molecular aspects of the Golgi stress response, as for example, the sensor molecule for the activation of the TFE3 pathway, are still unknown. Currently, the TFE3 pathway is activated mainly using pharmacological treatments, such as monensin and nigericin^3^. Our results revealed, for the first time, that the Golgi stress response is indeed activated under physiological conditions. In addition, the results here presented suggest that infections with DENV and ZIKV, or expression of recombinant NS1 may become additional experimental tools in the study of the Golgi apparatus and its stress response. Numerous are the examples of how the study of viruses had been rewarding to study the biology of cells.

## Materials and methods

### Cell cultures

C6/36 mosquito cells from *Aedes albopictus* (ATCC® CRL-1660™) were grown at 28 °C in Eagle's Minimum Essential Medium (EMEM) (ATCC® 30-2003™), supplemented with 5% fetal bovine serum (FBS) and 100 U/ml penicillin–streptomycin. Baby hamster kidney cells (BHK-21, ATCC® CCL-10™) were grown at 37 °C and cultured in Eagle's Minimum Essential Medium (EMEM, ATCC®) supplemented with 5% FBS and 100 U/ml penicillin–streptomycin. Monkey kidney epithelial cells Vero E6 (ATCC® CRL-1586™) were grown at 37 °C and cultured in Eagle's Minimum Essential Medium (EMEM, ATCC®) supplemented with 10% FBS and 100 U/ml penicillin–streptomycin. Crandell-Resse feline kidney (CRFK, ATCC® CCL-94) cells were grown at 37 °C in Eagle's Minimal Essential Medium with Earle's balanced salt solution, 2 mM l-glutamine, 1.0 mM sodium pyruvate, 0.1 mM nonessential amino acids, 1.5 g/l sodium bicarbonate, supplemented with 10% bovine fetal serum, 5000 U of penicillin, and 5 μg/ml of streptomycin. All cell lines were grown in a 5% CO_2_ incubator.

### Virus strains

DENV serotype 2, strain New Guinea and a Mexican isolate from *A. aegypti* of ZIKV, Asian genotype, were generously provided by M.Sc. Mauricio Vázquez, (Laboratorio de Arbovirus y Virus Hemorrágicos. Instituto de Diagnóstico y Referencia Epidemiológicos. InDRE, Mexico City) and propagated in C6/36 cells. DENV and ZIKV titers were determined by focus forming assays^18^. The feline calicivirus (FCV, strain F9) was propagated and titrated by plaque forming units assay in CRFK cells^31^.

### Drug treatment

Monensin disodium salt (M5273, Sigma-Aldrich®) was dissolved in ethanol to a concentration of 10 mM. Monensin is an inductor of the Golgi stress response and was used as positive control. Cells were treated with monensin at a final concentration of 5 µM in EMEM supplemented with 5% FBS, for 24 h. The conditions for non-toxic treatment of the different cell lines with monensin were determined previously, using cell viability MTT assays, according to the manufactures´ instructions (V13164, Themofisher).

### Virus infections

Confluent cell monolayers grown in 24-well plates (2 × 10^5^ cells per well) were infected for 2 h, using a MOI = 3 for DENV and ZIKV, and a MOI = 5 for FCV. After infection, the monolayers were washed three times with PBS to remove unabsorbed viruses, and infections allowed to proceed for 48 h for DENV or ZIKV, and 5 and 7 h for FCV. Afterward, cells were washed, fixed and stained for immunofluorescence.

### Plasmids transfection

Plasmids expressing recombinant DENV-NS1 and DENV-NS3 were kindly donated by Dr. Ana Fernández-Sesma (Icahn School of Medicine at Mount Sinai, New York). Recombinant plasmids expressing the transcriptional factor E3 (pTFE3-Myc) were kindly donated by Dr. Hiderou Yoshida (University of Hyogo, Japan). Plasmids were transfected into confluent monolayer of cells grown in 24-well plates using lipofectamine reagent Lipofectamine^TM^2000 (11668019, Invitrogen); each well was transfected with 1 μg of plasmid DNA and 2 μL of Lipofectamine in a final volume of 0.25 ml of EMEM w/o FBS. After 5 h of transfection, 0.25 ml of EMEM supplemented with 10% FBS were added per well. Cells transfected for DENV-NS1 and DENV-NS3 were fixed and stained for immunofluorescence at 24 h post transfection. Cells transfected with pTFE3-Myc were infected with DENV or ZIKV 24 h after transfection and the infection allowed to proceed for additional 48 h before fixing and staining for immunofluorescence. In addition, cells transfected with pTFE3-Myc were treated with monensin for 24 h, fixed and stained for immunofluorescence.

### Confocal microscopy

Confluent cell monolayers, grown in 24-well plates containing glass coverslips, were infected, or transfected as described above. At the indicated times, cells were washed once with PBS, fixed in paraformaldehyde 4% for 10 min and permeabilized with 0.1% Triton X-100 for 10 min at room temperature. Cells were stained for DENV-NS1 using Mab2B7, for DENV-NS3 using Mab 1ED8 (both Mabs a kind gift of Dr. Eva Harris, UC Berleley), for FCV-VP1 (SC65625, Santa Cruz Biotechnology), the Golgi complex was visualized using a commercial anti-GM130 antibody (GTX130351, GeneTex), and the TFE3 using a commercial anti-Myc antibody (GTX30518, GeneTex), as primary antibodies. As secondary antibodies, anti-mouse Alexa-488, anti-rabbit Alexa-568 and anti-goat Alexa-568 conjugated (donkey pre-adsorbed, secondary antibodies, Abcam) were used at 1:800 dilution in PBS. Nuclei were counter stained with DAPI (D9542, Sigma-Aldrich®). Coverslips were mounted in Fluoroshield™ with DAPI (F6057, Sigma-Aldrich®). The slides were examined using a Zeiss LSM 700 confocal microscope.

### Translocation of recombinant transcriptional factor E3

Percentage of translocation of TFE3 from the cytoplasm to the nucleus was calculated through the comparison of the median fluorescence intensity (MFI) in the nucleus versus the cytoplasm (MFI nucleus area/MFI cytoplasm area × 100) in each of the experimental conditions. At least 10 cells per condition were examined and the results analyzed using test one-way ANOVA. The images were processed with Zen Blue edition 2.6 software and the results were plotted with the GraphPAD Prism version 8.01.

### rRT-PCR assays

BHK-21 cells were infected with DENV or ZIKV at an MOI = 3 and harvested at 24 hpi. Mock infected cells or cells treated with monensin 5 µM were used as negative and positive controls. Total RNA was isolated with Rneasy Kit (Qiagen Cat. No. 74004) according to the manufacturer’s procedures. A total of 0.1 µg of total RNA was used to determine the relative quantification of each target using the housekeeping GAPDH gene expression as reference. One step RT-PCR was carried out using the QuantiTect probe RT-PCR kit (Qiagen, Valencia, CA) with 500 nM forward and reverse primers and 50 nM labeled probes (GOLGA2-FAM, GCP60-FAM and GAPDH-VIC TaqMan). Detection primers and probes were designed for each target (BHK_GOLGA2_fw CTGCGGCCAAGAAAAAGTTGAG, BHK_GOLGA2_Rv TGCCATGCTAGTGAGGTCAG, BHK_GOLGA2_P ACCAGAGGACGCGCCCACAGA, BHK_GCP60_fw GCGCTGCGCTTCTTCAAAATA, BHK_GCP60_Rv CTGCCCATTCTCTCCTCCTAT, BHK_GCP60_P CCAGACACTTGCCCTGAAGTTGGA, Hs_GAPDH_F CATGGCACCGTCAAGGCTGA, Hs_GAPDH_R ACGTACTCAGCGCCAGCATC and Hs_GAPDH_P TCCAGGAGCGAGATCCCTCCA). Cycling conditions for all targets were 50 °C for 10 min, 95 °C for 2 min, 45 cycles of 95 °C for 30 s, and 58 °C for 30 s and 68° for 30 s. Amplification was done in a StepOne real-time PCR device from Applied Biosystems (Applied Biosystems, Foster City, CA), and results were analyzed using StepOne software v2.3. Each assay was performed in two biological replicates with three technical replicates, and each assay included no-template negative controls. Results were expressed as relative expression levels of target mRNA calculated as RQ (2^−ΔΔCt^) using of GAPDH mRNA levels of expression as housekeeping reference. Statistical analyses were carried out using GraphPad Prism, version 6.01, software.
